# A Toolkit for Co-Designing towards Community-Based Active Ageing: Lessons Learned during Development

**DOI:** 10.3390/ijerph192315591

**Published:** 2022-11-24

**Authors:** Gubing Wang, Dena Kasraian, Carlijn Valk, Yuan Lu, William Hurst, Marielle Jambroes, Pieter van Wesemael

**Affiliations:** 1 Department of Built Environment, Eindhoven University of Technology, 5600MB Eindhoven, The Netherlands; 2 Department of Industrial Design, Eindhoven University of Technology, 5600MB Eindhoven, The Netherlands; 3 Department of Informational Technology, Wageningen University and Research, 6706KN Wageningen, The Netherlands; 4 Department of Public Health, University Medical Center Utrecht, 3584CX Utrecht, The Netherlands

**Keywords:** behaviour change design, community-based design, ergonomics in ageing, guidelines, design tool, interdisciplinary collaboration, participatory design, hackathon, older adults, rapid review

## Abstract

It is increasingly recognized that community-based interventions for active ageing are more lasting and effective, yet the tools and methods for developing these interventions are lacking. This study investigates how to co-design community-based active ageing with older adults via the development of a toolkit to support this goal. Rapid reviews were conducted to understand (i) the effective behavioural change techniques for older adults, (ii) how to co-design with older adults for community-based interventions, and (iii) how to design tools for behaviour change that are easy to use. These reviews served as the foundation for developing a toolkit to support the co-design of community-based active ageing, which was evaluated during an interdisciplinary hackathon with older adults. Quantitative data from the surveys suggested that the confidence levels of students in developing interventions for health behaviour change and in co-designing with older adults increased after the hackathon, and the enjoyment of participating in the hackathon and of using the toolkit were statistically significant factors influencing this increase. Qualitative data from interviews and observations revealed how the toolkit was (un)used by the participants and what aspects of the toolkit can be improved. We encourage future researchers and practitioners to apply and adapt our research findings to the communities of older adults that they are working with.

## 1. Introduction

The vulnerability of older adults (OA) was further threatened during the COVID-19 pandemic, and researchers are calling for a re-evaluation of the strategies for promoting active ageing [1]. Since the World Health Organisation proposed a policy framework for active ageing in 2002 [2], the definition of active ageing has been broadened from the concepts of economic engagement and involvement in physical exercises to engagement in life in general [3]. The emerging literature on behaviour change has placed increasing emphasis on community involvement [4,5]. Community-based interventions were mainly created to foster healthier lifestyles and inclusive practices, which have been found to be able to sustainably engage residents cognitively, affectively, and behaviourally [6]. One of the most urgent and important research question is how to facilitate the process of designing for community-based active ageing. The Behavioural Change Wheel (BCW) developed by Michie et. al. offers a systematic way to develop interventions for health behaviour change [7]. This framework was developed based on a systemic review of previous frameworks for behaviour change interventions. At the centre of the BCW is a “behaviour system” that involves three essential conditions for behaviour change, namely, capability, opportunity, and motivation. Behaviour change techniques (BCTs) are part of the framework that connects the theoretical understanding of behaviour change to concrete guidelines for designing behaviour change interventions. The BCW was used to characterise two national interventions for health behaviour change, and based on its reliable outcomes, it has been widely applied in practice ever since. However, some BCTs proposed by the BCW that are effective for young adults may not be effective for OA. For example, as people age, their executive function decreases [8]; additionally, in later life, people’s life goals become more focused on maximising meaning and positive emotions, while improving health is associated with delayed future payoffs, which are of less concern to OA [9].

Since OA are a highly heterogeneous group, for example, in terms of their health status, experiences, abilities and interests, we have adopted the concept of “situational elderliness”, as suggested by Brandt et al. [10]. Social and cultural gerontologists propose that old age is a social construct defined by everyday practices and encounters [11]. According to Brandt et al., rather than recruiting individuals based on their chronological age, it is more likely that the right group of people will be recruited by approaching communities of everyday practices, where “seniors are skilfully enacting everyday practices as seniors” [10]. This is because people might experience situated elderliness as they grow older, for example, in the case of gardening, only some tasks (e.g., digging, weeding) are difficult for some OA while they are able to handle the other tasks. Like gardening, there are many other daily activities that OA might need help with, and their need to belong to a community might therefore increase. This need has increased further due to the trend whereby OA and their younger relatives live further apart from each other, along with other societal developments.

Moreover, including users and other stakeholders in the design process can lead to designs that better meet their needs and preferences [12]. It has been proposed that co-design should be integrated into the intervention development process for ethical reasons and to develop a sense of ownership to foster sustainable behaviour change [13]. A growing body of work has explored how to co-design with communities in the public health domain [14,15]. However, there are few guidelines on how to co-design with OA communities. The recent research on co-designing interventions for OA falls into three categories. In the first category, even though OA are the end-users of an intervention, the intervention is co-designed among experts only (e.g., healthcare professionals), and tools have been developed to facilitate this kind of co-design (e.g., [16]). The second category includes studies that report interventions that are co-designed with OA, although there is no description of how the co-design was undertaken (e.g., [17,18]). The last category in the literature includes studies that report the details of how they engaged OA in the co-design or refer to the co-design methods applied (e.g., [19,20,21,22]).

Within the last category, a number of the tools used for facilitating the co-design process have been described and evaluated. Here, we adopt a broad definition of “tool”, as in the Cambridge Dictionary, which refers to “something that helps one to do a particular activity”; thus, the form of the tool could be tangible or intangible (e.g., framework). We also distinguish between “tools” and “materials”. In comparison to tools, materials are more generic and can be used in co-design sessions with different design goals (e.g., LEGO, Play-doh). Tangible tools are usually used by the participants during the co-design sessions, while intangible tools are mainly used by designers to plan the co-design session (e.g., [20,23]). Tangible tools can be grouped into three types based on their format, namely, digital, analogue, and hybrid. The tools used for increasing the digital literacy of OA include all three format types (e.g., card-sets [24], sensor kits [25], interactive dice [26]) while the tools used for facilitating collaboration and expression are usually analogue and emphasize the importance of being tangible to spark one’s creativity [27].

In terms of the purpose of the tools, there are two main goals. The first is to help OA to become familiar with technology or to increase their digital literacy so that OA have enough knowledge to use the technology in the future or are inspired to be more creative by knowing what technology can do [25,28,29]. The second goal focuses on facilitating OA to express themselves during workshops, for example, their ideas and emotions, in an easier way as compared to focus groups or other traditional user research methods [27]. All of these tools aim to spark creativity within OA. Except for Gooch et. al., who explored co-designing with OA for increasing physical activities [27], all the other tools mentioned above were not utilised for addressing behaviour change. Gooch et. al. also reported that not all OA used the storyboard template during the co-design session, which is an interesting finding that is worth investigating. Gooch et. al.’s work is also the only study that explicitly designed an intervention at the community level [27]. The existing tools for facilitating the design of behaviour change were mainly created for designers and evaluated by designers [30,31,32,33]. Whether these tools can be used to co-design with target users is not yet known. Our previous work on ergonomics in ageing reviewed the differences in the capabilities of young adults and OA [34]; this could provide a foundation for the development of design tools for behaviour change that are user-friendly for OA.

The aim of this study was two-fold. First, via a rapid literature review, we identified (1) the BCTs that are effective for behaviour change in OA, (2) methods and tips for co-designing with OA communities, and (3) methods and tips for developing easy-to-use tools for designing behaviour change. Second, based on the knowledge gained above, we developed a toolkit for co-designing with OA on community-based active ageing, which was evaluated in a hackathon on supporting OA with group gardening. Specifically, students and OA were recruited, a quantitative survey was conducted to investigate if using the toolkit has an effect on the confidence level of participants in co-designing behaviour change interventions, and qualitative feedback was gathered on how to improve the toolkit further.

The structure of the paper is as follows. In Section 2, we describe the methods used for the rapid review and for the toolkit development. When evaluating the toolkit in the hackathon, we conducted pre-post surveys, observations, and semi-structured interviews for triangulating our findings. Then, in Section 3, we report on the findings from the rapid reviews, the final version of the toolkit, as well as the quantitative and qualitative results of the toolkit evaluation. We then provide a discussion of our findings from the rapid reviews, the design implications for toolkit development, and the limitations of this study (Section 4), followed by some remarks and indications for future work (Section 5).

## 2. Materials and Methods

**Figure 1:**
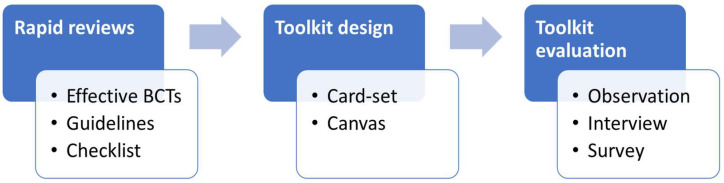
Overview of the study flow.

The key steps in this study and the main outcomes within each step are summarised in Figure 1, which are explained in the following subsections. The study protocol was approved by the Human Research Ethics Committee of the Eindhoven University of Technology. All participants filled in an informed consent form before the hackathon.

### 2.1. Rapid Reviews

For the first research aim, we conducted a rapid review to extract evidence and learnings from previous studies on each of the following three aspects: (i) effective behavioural change techniques for OA, (ii) how to co-design with OA for community-based interventions, and (iii) how to design tools for behaviour change that are easy to use. In a rapid review, sources are limited due to the time constraints of searching; however, transparent and reproducible search methods are still used [35]. To date, there is no typology of the methods for rapid reviews, and the main difference between our rapid review and a systematic review is that our review is limited to the literature published in one database. This method was used due to the urgent need to develop the toolkit for co-designing with OA for community-based active ageing. A literature search was conducted in early December 2021. The database Scopus was searched. All the relevant articles published with available full text were collected, and the duplicates were removed. No hand search was performed. In Table 1, the three rapid reviews are summarised in terms of the “goal of review”, “review type”, “search terms”, and “studies included (n)”. The flowchart depicting each literature search can be found via the Open Science Framework (OSF) link: https://osf.io/g6479, accessed on 10 October 2022.

The **effective BCTs** identified were then summarised and categorised based on the stages of behaviour change [36]. According to health psychology, people usually go through several stages starting from the moment they start thinking about changing their behaviour to the moment that they have changed it permanently. To facilitate the transition from one stage to the next, the goal of the design is different, and includes “raising awareness”, “enabling”, “motivating” and “fading-out” [36]. An explanations of the four categories can be found via the OSF link: https://osf.io/g6479 (accessed on 10 October 2022). The lessons learned from the second review were extracted to form the **guidelines** for co-design with OA communities. The insights gained in the third review were summarised and combined with our previous findings in Ergonomics in Ageing [34] to form the **checklist** for developing tools for co-design with OA for behaviour change.

### 2.2. Toolkit Design

The toolkit was then developed based on the **effective BCTs** and the **checklist**, while a hackathon was planned based on the **guidelines** and the toolkit was evaluated in the hackathon. In this paper, we focus on the evaluation of the toolkit, and the evaluation of the hackathon is reported elsewhere [37]. The design process of the toolkit is described in the following paragraph. The main reason why we evaluated the toolkit in a hackathon is that the hackathon offers a real-life challenge for co-designers; additionally, the limited resources and time encourage participants to think creatively and collaborate effectively. Moreover, given the stage of development of our toolkit, a hackathon could help us to quickly evaluate our toolkit before the next iteration. In contrast, a design project can last for a number of weeks, so it is not cost-effective for researchers to undertake observations throughout, especially when there are several teams. Evaluating the toolkit in a design project might only be worthwhile after we improved our toolkit based on the hackathon.

The target group for the toolkit was first determined to be anyone interested in co-designing for community-based active ageing. The user does not need design training to use the toolkit. The **effective BCTs** were fed into the content of the toolkit. Specifically, the toolkit was composed of two parts, a card-set and a “canvas”. The card-set allows participants to explore and become familiarised with the effective BCTs and think of ideas related to the hackathon challenge based on each BCT. The aim of the canvas is to guide participants through the design process, from learning about behaviour change models, identifying resources in the community, generating concepts, and finally, evaluating concepts. The card-set was intended to be applied in the concept generation stage (i.e., step 3 on the canvas). The **checklist** was applied to define the design requirements of the toolkit so that it was inclusive to OA. Before the hackathon, feedback about the toolkit was sought from other hackathon organisers and potential hackathon participants (i.e., students and OA representatives, based on which the toolkit was iterated five times before the pilot study. After the pilot study, there was one more round of iteration to prepare for the hackathon.

### 2.3. Toolkit Evaluation

The hackathon was organised together with a senior centre, Ontmoet & Groet (O&G), in the Netherlands. The mission of O&G is to help OA living in the neighbourhood to stay socially, physically and mentally active. The hackathon was interdisciplinary with students (i.e., under Bachelor, Master, and PhD education) from four disciplines (Industrial Design, Informational Technology, Public Health, and Built Environment) across three universities in the Netherlands. The hackathon was hosted on the 4 May 2022 from 9:00 to 18:00 in a meeting room at a nursing home that is a 5 min walk from O&G. The hackathon involved 16 students and nine OA participants. The 16 students were divided into four interdisciplinary teams seated at four tables. A toolkit was placed on each table along with generic materials for co-design (e.g., cardboard, Play-doh, post-its). The participants recruited from Public Health were medical students who had experience in interacting with OA.

We would like to note that OA were only involved in parts of the hackathon process to prevent them from tiredness. Two co-design sessions with OA were scheduled during the day. Four OA participated in the morning co-design session from 10:30 to 12:00 and five OA participated in the afternoon co-design session from 14:00 to 15:15. They were matched to each team by the CEO of O&G with two OA participants allocated to one team in the afternoon session. The CEO of O&G knew the OA participants personally, and he made the allocation based on observations of the students during the hackathon to create the most productive group dynamics.

During the hackathon, qualitative data regarding the usage of the toolkit was collected in terms of (1) if participants asked questions about the toolkit, (2) if participants explained to each other how to use the toolkit, and (3) if participants stopped using the toolkit. Four field researchers, one from each discipline, conducted participatory observation as the coaches during the hackathon, and each noted down observations on a formatted observation template. Group interviews were conducted with the OA participants immediately after the co-design sessions at the venue of the hackathon (*n* = 9). Two field researchers conducted interviews in Dutch on the experiences of OA during the hackathon. A follow-up individual interview was conducted with students (*n* = 12) from one day to two weeks after the hackathon during which descriptions of their experience of using the toolkit were obtained. For the quantitative data, a pre–post survey was introduced to collect the confidence levels of students in designing for behaviour change and in co-designing with OA. Fourteen students filled in the survey before the hackathon and one student did not complete the survey after the hackathon in time (*n* = 13). The post-survey also contains a validated creativity support index to collect the experiences of students with the toolkit for triangulation with the qualitative data [38]. We selected the creativity support index because it is designed based on concepts and theories from creativity research and specifically for evaluating creativity support tools, which aligns with the goal of our toolkit.

In summary, we adopted a mixed methods approach to evaluate the developed toolkit. The quantitative data helps us to understand the effect of using the toolkit on the confidence levels of participants in co-designing interventions for health behaviour change, which is more generalisable. Although the qualitative data is more contextualised, it helps us to gain a deep understanding of the experiences of participants when using the toolkit, which enables us to improve the toolkit. The quantitative and qualitative data complement each other to help us thoroughly answer our main research question. Based on the work of Doyle et al. on mixed methods research [39], both types of data were collected concurrently and integrated with equal weighting during the interpretation stage.

## 3. Results

In this section, we report on the findings from the rapid review, the final version of the design toolkit before the hackathon, as well as the quantitative and qualitative insights on the toolkit that were collected from the hackathon.

### 3.1. Rapid Review Findings

The findings from the rapid review were summarised into “the effective BCTs”, “the co-design guidelines” and “the tool development checklist”, which will be introduced in detail below.

#### 3.1.1. The Effective BCTs

The effective BCTs that were identified are shown in Table 2. No effective BCT fits in the “fading-out” category, several BCTs can be considered as both “enabling” and “motivating” for one to change behaviour. The definition of “OA” varied across the studies, and half of the studies focused on interventions promoting physical activities. It is worth noting that one study concluded that BCTs might be less suitable for OA [40], while the other studies reported the effective BCTs [41,42,43,44,45]. Therefore, the evidence for effective BCTs is heterogeneous. The most-mentioned BCT is “problem-solving” (in four studies); followed by “goal setting”, “action planning” and “social support” (each in two studies).

Only four studies reported on the effectiveness of the interventions, while two of them reported that the effectiveness was limited, the other two studies reported that the interventions were effective. Among the latter two studies, one study reported that the effectiveness of the interventions lasted longer than one year while the other study reported that the effectiveness of the interventions beyond one year was unclear. An elaborated version of Table 2 (with explanations and examples) and more details regarding all the studies included in the review can be found via the OSF link: https://osf.io/g6479, accessed on 10 October 2022.

#### 3.1.2. The Co-Design Guidelines

The guidelines summarised for co-designing with OA for community-based interventions can be found in Table 3. The reviewed studies revealed three approaches to co-designing with OA for community-based interventions. The first approach is to co-design only with OA for interventions to be used by all the OA in the community [9,46]; the second is to co-design with OA and other generational groups (e.g., children, and younger adults) for interventions to facilitate intergenerational bonding in the community [47,48]. The third way is to co-design with OA and local organisations (e.g., local hospitals, local NGOs, and municipalities) for community-based services for OA from these organisations [49]. These guidelines are categorised as “involving OA”, “fostering intergenerational bonding”, and “connecting local organisations”. More details about all the studies included in this review can be found via the OSF link: https://osf.io/g6479, accessed on 10 October 2022.

#### 3.1.3. The Tool Development Checklist

The checklist for developing tools for co-designing with OA for behaviour change can be found in Table 4. The reviewed studies each report a tool/toolkit for designing behaviour change. Three of the four studies used a card-set form for the tool [31,32,33]. The other study created a diagram of their developed model as part of the toolkit and the rest of the toolkit was not described [30]. Two studies explicitly reported the theories that supported their choice of tools [31,32]; while the other two studies reported the disciplines from which the tools were derived and did not specify the theories behind them [31,33]. All studies evaluated their tool/toolkit with either design students or design professionals. Some evaluations are more thorough than others. None of the tools or toolkits were evaluated in a co-design situation for designing behaviour change. This is the main limitation of these tools and toolkits if they are applied to co-designing with OA for behaviour change. The other limitation of two of the tools reviewed is that they are focused on designing interactive technologies [31,32]; while technology broadens the solution space for design, it is not an essential element in all the interventions. The studies reported the useful features of their tools and toolkit in different levels of detail, while one study reflected on areas for improvement regarding the current features based on the evaluation feedback [32]. More details about all the studies included in the review can be found via the OSF link: https://osf.io/g6479/, accessed on 10 October 2022.

By integrating the findings from this rapid review and our previous work on ergonomics in ageing, a checklist was developed that consisted of questions and recommendations. The types of questions were divided into “would you like…” (offer developers a choice) and “have you…” (remind developers about important features). By “developers”, we mean the researchers and designers who will develop tools/toolkits. There is no particular order to using the checklist, and we encourage developers to read through the checklist in detail at the beginning of the development process to be aware of what features could be useful for the tools that they are developing.

### 3.2. The Toolkit

**Figure 2:**
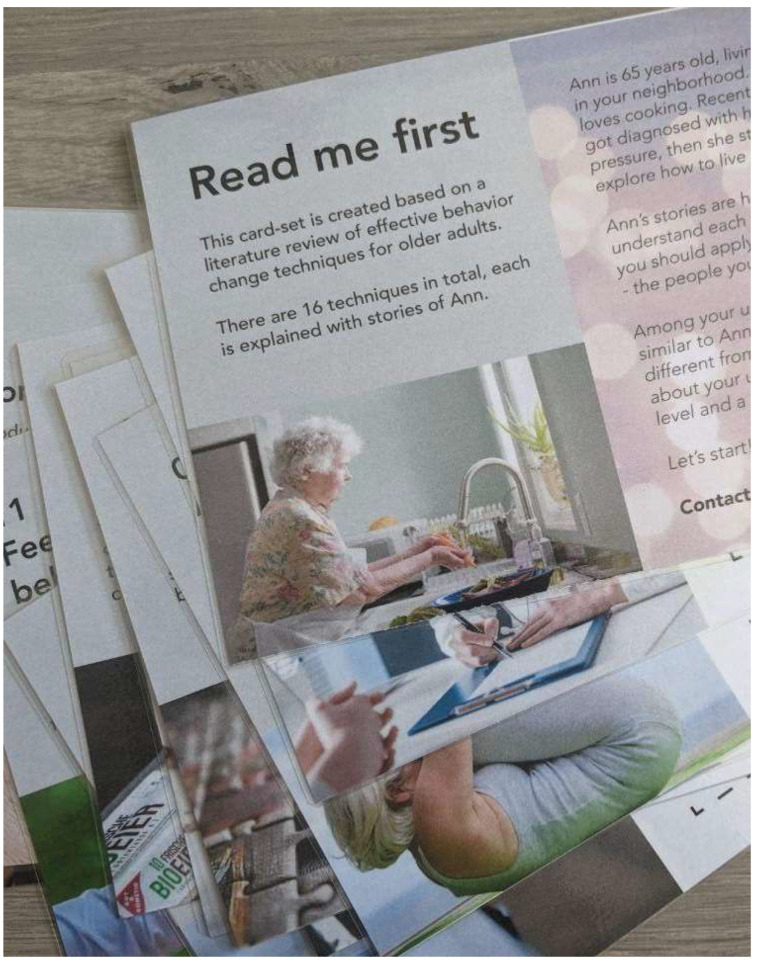
The card-set of the toolkit.

**Figure 3:**
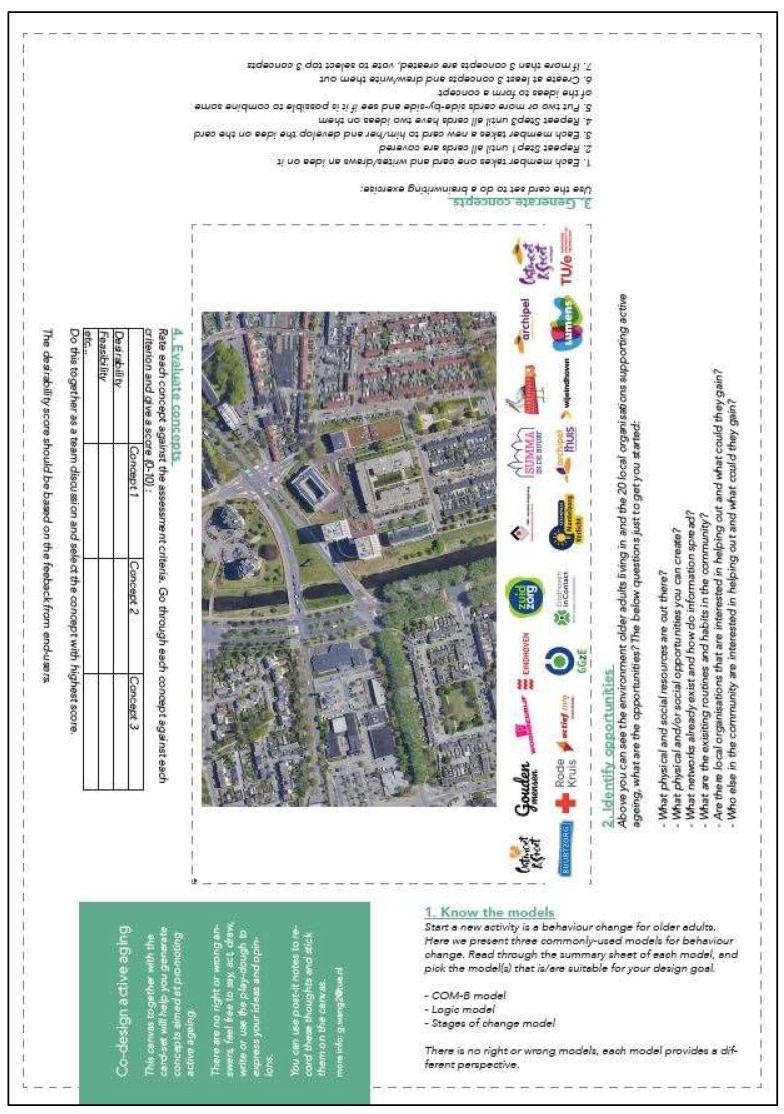
The canvas of the toolkit.

**Figure 4:**
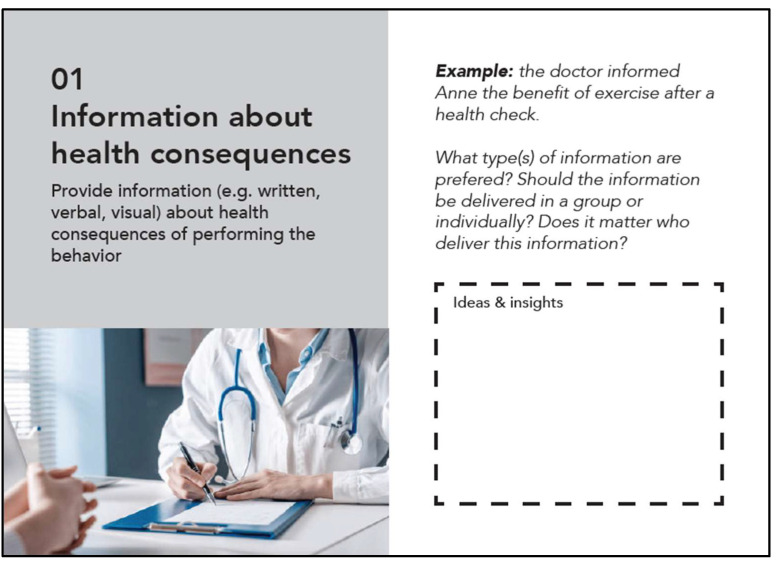
Example of a BCT card.

The toolkit contains a card-set (17 cards of size A5) and a canvas (size A1). The aims of the toolkit and how it is intended to be used can be found in the Methods section. A snapshot of the card-set and canvas can be found in Figure 2 and Figure 3 respectively. To ensure the user-friendliness of the card-set, the effective BCTs were grouped into sets of 16 cards based on their similarities. The intro card of the card-set introduces a fictional character Anna and her challenges in helping users build empathy, and the remaining cards each focus on one effective BCT for OA. “Users”, refer to the users of the toolkit. Each BCT card consists of a definition of the BCT, an example of Anna applying the BCT, an illustration photo, several stimulating questions, and a blank space for users to record their ideas and insights. An example of the BCT card is shown in Figure 4. One side of the card is in English, and the other side is in Dutch (the native language of the OA participants). For the canvas, an Open Street map of the neighbourhood in which OA live was positioned in the centre, together with the names of local organisations that support active ageing. The description of each design stage was laid out facing towards the four sides of the canvas, and the intention was to let participants put the canvas on the table, sit around it, and take turns to lead in each design stage. The canvas is in English only because it is intended to be used by the student participants, who are fluent in English and some of whom cannot speak Dutch. We assumed the students would easily explain each design stage to OA via conversations during the hackathon. The full-size version of both the card-set and the canvas can be found via the OSF link: https://osf.io/g6479, accessed on 10 October 2022.

### 3.3. Quantitative Insights from the Evaluation

**Figure 5:**
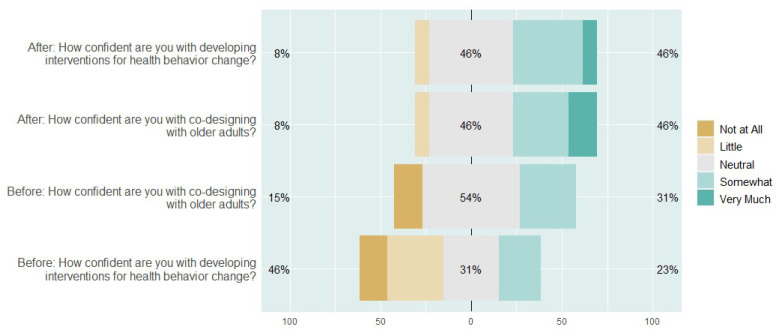
Confidence levels before and after the hackathon ordered by Strongly Disagree (from not at all to very much).

The quantitative analysis of the surveys indicated there was an increase in the confidence levels of students in regard to the design of health behaviour change and in co-designing with OA. The confidence levels before and after the hackathon are displayed in Figure 5, which depicts a Likert-scale score ranging from “Not at all” (1) to “Very much” (5), with the *x*-axis indicating the percentage of respondents selecting a grading. The *y*-axis presents two questions that were posed to participants before and after the hackathon: (1) *How confident are you with co-designing with OA*? and (2) *How confident are you with developing interventions for health behaviour change*? Notably, no participants selected 5 for “Very much” before the hackathon for either question, potentially indicating a level of hesitation in regard to the co-design process. The percentages in the figure refer to the positivity compared to the negativity toward the questions. For example, for the first question, ‘After: How confident are you with developing interventions for health behaviour change?’, 8% answered negatively, 46% were neutral and 46% said they were more confident.

Based on visual inspection, the overall confidence levels increased, with 2 participants selecting 5 (Very much) for question 1 (*How confident are you with co-designing with OA*) and 2 participants selecting 5 for question 2 (*How confident are you with developing interventions for health behaviour change*).

Further investigation was conducted on the influencing factors for the measured change in confidence in *developing interventions for health behaviour change* before and after the hackathon. Six predictor variables were considered, which related to six questions from the survey (the first question was designed by the authors and the other five were from the creativity support index for evaluating the toolkit), with the outcome variable being the level of confidence in *developing interventions for health behaviour change* post-hackathon. This outcome variable was the focus of the statistical analysis because, as indicated in Figure 5, the question ‘*How confident are you with developing interventions for health behaviour change?*’ displays the largest change in confidence from the post-to-pre hackathon compared to confidence in co-designing with OA. These questions are listed (with their corresponding abbreviations) in Table 5. For each question, the respondent was able to select a Likert-scale answer of 1–5 (1 being the lowest, and 5 the highest).

To test our hypothesis, a multiple linear regression model was employed, from which the *t* and *p*-values were calculated by the car library in R (see Table 6). The multiple linear regression model was selected as it caters for numerous predictor variables. The algorithm is outlined in Equation (1) in matrix form and includes ε and β, which refer to the vector of errors and coefficients, respectively. **Y** is the vector of the values of the outcome variable, i.e., in this study, this was the confidence level scores post-hackathon. Finally, **X** refers to a matrix of values of the predictors.

**Y** = **X**β + ε (1)

VanVoorhis and Morgan et al. suggest that for regression-based assessment (with >6 predictor variables), one should have a minimum of n = 10 participants [50]. We have n = 13 participants for the evaluation process; therefore, a linear regression model is a suitable approach. Both the *p* and *t*-values are measures of statistical significance. A *p*-value of <= 0.05 and a *t*-value of >2 were defined as indicating significance.

**Figure 6:**
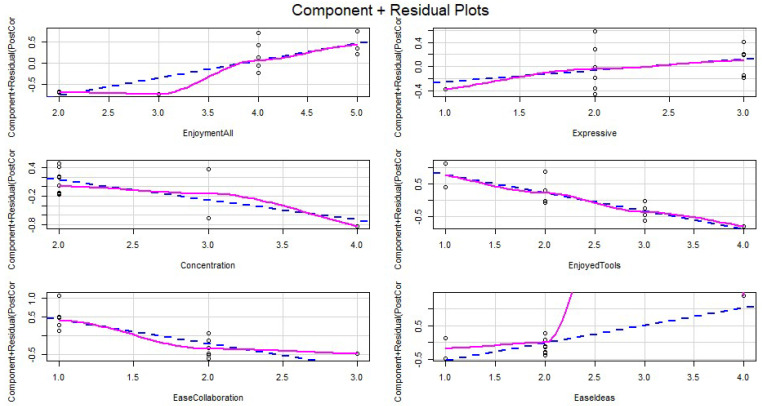
Linear regression component and residual plots.

The component-plus-residual plots for all predictor variables are displayed in Figure 6. The blue dashed line indicates where the linear relationship should be, and the purple continuous line refers to the estimated linear relationship. Ease_Ideas appears to be the least accurate (when compared with the other 5) as the purple line diverges significantly from the blue linear relationship.

The results indicate that participant’s enjoyment of the overall process (Enjoyment_All, *p* = 0.0436 and *t* = 2.684) and enjoyment of using the tools (Enjoyed_Tools, *p* = 0.028 and *t* = −3.062) were statistically significant factors that affected confidence levels post-hackathon. The negative *t*-value for Enjoy_Tools indicates a reverse directionality of the effect; this has no bearing on the significance. The results indicate that enjoyment of the event has a positive effect on confidence levels.

**Figure 7:**
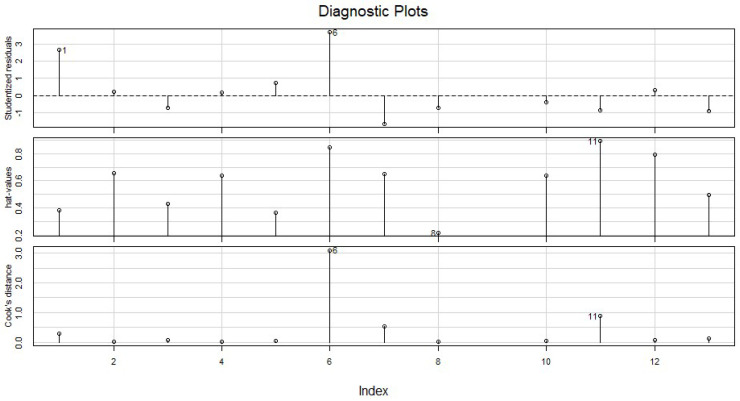
Diagnostic plots.

The multiple linear regression analysis produces a multiple R-squared value of 0.847 (with an adjusted R-squared of 0.662) and a residual standard error of 0.464. In Figure 7, the diagnostic plots display the 13 participants on the *x*-axis, with the influence values (cook), outliers (studentised) and leverage (hat) on the *y*-axis, where a Cook’s distance (*d*-value) > 1 is indicative of influence. The plot indicates that participant 6 had an influence on the findings with a *d*-value of 3 (where >1 indicates influence) and is also a clear outlier in the studentised graph when visually compared to the others. Investigating the survey responses of the individual may offer insight into the reason for the high outlier score. In the pre-survey, the participant had also previously worked on projects related to interventions (e.g., design/activity) for health behaviour change and worked with OA before the hackathon. The individual’s confidence level was high both before and after the event while this individual responded to other questions as follows: Enjoyment_All: 4, Expressive: 2, Concentration: 2, Enjoyed_Tools: 1, Ease_Collaboration: 3, Ease_Ideas: 2. An indication of the higher outlier score visible in the diagnostics plot (Figure 7) may be due to the participant’s selection of high scores for confidence and enjoyment, yet relatively low scores for all other categories. In this case, it could be that the confidence is due to the participant’s previous experience and enjoyment of the topic rather than the event itself.

### 3.4. Qualitative Insights from the Evaluation

The qualitative insights were generated based on the analysis of observations, interviews with OA during the hackathon, and follow-up interviews with students after the hackathon. Three teams used the toolkit while team 1 did not use it. When asked, members from team 1 reported that they obtained enough information and ideas from talking with OA. One member of team 1 did scan through the card-set and canvas at the very beginning of the hackathon. During the individual interview, he reflected that the information provided by the toolkit was “not sufficient for the hackathon”. The hackathons he had attended before always included young adults with programming backgrounds as participants. When comparing the information to that he had received from previous hackathons, he felt he was “missing a large amount of information to process” in the toolkit.

We observed that the other three teams asked questions about the toolkit during the hackathon, which focused on explaining information provided by the canvas (e.g., behaviour change models, logos of local organisations, meanings of certain terms). These three teams used the toolkit after co-design sessions with OA and it was the design students who guided the students from other disciplines on using the toolkit. Team 2 and 4 used the card-set and canvas briefly. Instead of following the design process as shown on the canvas, they used the canvas as a checklist to ensure they had covered all the points; for the card-set, they formed the concepts first and chose a number of cards from the card-set that supported their concepts. Our observations revealed that team 3 used the toolkit the most thoroughly among all teams. They followed the steps described on the canvas and tried to use as many cards as possible for concept development.

All the teams mentioned that the hackathon schedule was tight and team 3 did not have enough time to complete all the stages as listed on the canvas. Similarly, many participants found there was too much information to process. We reflected on this and generated procedural knowledge on how to introduce a toolkit for evaluation, which can be found below in the discussion section.

With regard to the card-set, the main points of improvement can be categorised into three groups: (1) less technical language, (2) more visual information and less text, and (3) removing hard-to-apply BCTs. First, despite several rounds of iterations to ensure the BCT was easier to understand, three participants commented that the “examples are sometimes confusing” and “the card-set causes confusion”. Second, three participants stated that they would like the card-set to have “more visuals” with “less text” and “fewer examples”. One participant remarked that the examples provided in the card-set could bias the creativity of the team. Third, two participants found the BCT “self-reward” difficult to apply externally since they thought “a user would decide it by himself”.

With regard to the canvas, the main points of improvement can be categorised into three groups: (1) more visuals and less text, (2) smaller size, (3) a close-up map of the implementation location. First, four participants reported that they would like to see more visuals and less text on the canvas. The stages described on the canvas “look the same visually” and “more colours or pictograms” could “add structure” to it. Second, two participants found the size of the canvas inconvenient. The canvas was designed to be large enough to be put on the table so participants could sit around it, yet “other tools were covering it most of the time” and it was “hard to read all the parts” given its size. Third, since the goal of the hackathon was to develop an intervention for group gardening that will be implemented in the senior centre, two participants reported that they would like to know more about the “physical environment of the location where the intervention will be implemented”. Even though all the participants visited the senior centre in person during the lunch break, they thought that a close-up map of the implementation location could be helpful.

Interestingly, the opinions on the toolkit were similar within each team. For team 2, the repeated topics were (1) more visuals and less text and (2) less technical language; for team 3, the repeated topic was the lack of time available to use the canvas; for team 4, the repeated topic was hard-to-apply BCTs. This might be because the participants in the same team shared the same experience when using the toolkit and they influenced each other’s opinions while using the toolkit.

When categorised according to disciplines, the feedback from the Industrial Design participants was the most homogenous—four out of five participants mentioned that they would like the toolkit to be more visual and physical and have less text. The feedback from the participants of the other three disciplines was more heterogeneous.

All OA participants had positive experiences during the hackathon. They found that the students were “attentive, respectful and collaborative”; some of them did get confused about the location of the hackathon since it was not hosted at a place that was familiar to them. Many of them mentioned that they would like to get updated on how their ideas are taken to the next step of the research. One OA interacted with the canvas and used it as a tool to tell students how she tried to find the venue. The students noted that it was nice to see the canvas act as an icebreaker.

Each of the four teams generated one final concept during the hackathon. At the end of the hackathon, each team pitched their concept for 5 min to a group of judges. Seven of the judges worked in local organisations that promote active ageing and one judge has researched how to design for active ageing during her PhD. All judges gave the highest score to team 3. Four judges gave the second-highest score to team 4, two judges gave the second-highest score to team 2, one judge gave the same second-highest score to both teams 2 and 4, and one judge gave the second-highest score to team 1. We will report the details of the final concepts in our future work [37].

## 4. Discussion

The main findings of this study can be categorised into two layers. In the first layer, we identified effective BCTs for OA, extracted a guideline on how to co-design with OA communities, and formulated a checklist for developing tools that support co-designing with OA for behaviour change. In the second layer, a toolkit was developed based on the above insights and evaluated in a hackathon where students formed interdisciplinary teams to co-design with OA on a group gardening intervention for community-based active ageing. The toolkit was found to increase the confidence levels of the participants in co-designing interventions for behaviour change, and areas of improvement for the toolkit were identified.

### 4.1. Reflection on Effective BCTs

Regarding the effective BCTs, even though Zubala et. al. explicitly concluded that “BCT might be less suitable for OA”, they argue that “environmental and social supports” are “motivators more meaningful to them” [40]. We interpret this study to imply that some BCTs are less suitable for OA, such as goal setting and action planning, as they rely on one’s executive function and long-term vision (short-term pain for long-term gain). Some BCTs do acknowledge the importance of “environmental and social supports” for OA, such as “adding objects to the environment”, “social reward”, “social support”, and “restructuring the physical environment”. Interestingly, “goal-setting” and “action planning” have been mentioned in two reviewed studies as effective BCTs for OA. Even though the target group of Zubala et al. was community-dwelling people over 50 years old, we cannot guarantee that the sample in their review had lower executive functions and more of a living-in-the-moment attitude than the samples from other reviews. Many factors can affect the effectiveness of BCTs, such as health conditions, culture, and available resources. Therefore, we advise future researchers to use the list of effective BCTs for OA as a starting point and improvise based on insights gained during the process of co-designing with OA communities in their projects. 

### 4.2. Reflection on Co-Design Guidelines

Regarding how to co-design with OA communities, the guidelines are based on six studies that satisfied the inclusion criteria, which is a limited number. As more studies on this topic are undertaken in the future, these guidelines could be updated with state-of-the-art insights and advice. We see the guidelines as a starting point to prepare designers to co-design with OA for community-based interventions. Designers can choose to only involve OA in the community, or also include younger generations and/or local organisations. Some of the guidelines are more intangible (e.g., mindset) while others are more tangible (e.g., providing visual materials that OA can relate to). We have made the tangible part of the guidelines more concrete by providing a checklist for toolkit development. Especially in designing for behaviour change, the designers may be introduced to behaviour change theories for the first time; how can they prepare an easy way to introduce these theories and techniques to OA? We will discuss this checklist in the section below.

### 4.3. Reflection on the Checklist for Toolkit Development

This checklist was developed based on a limited number of studies, the majority of which focused on card-set. Therefore, similarly to the guidelines, we suggest that this checklist should be updated based on the development of future tools/toolkits. Furthermore, we acknowledge that more insights on how to create user-friendly design tools could be gained by reviewing studies on design tools and toolkits in general. However, we would like to keep this review more focused on behaviour change and we uncovered specific advice related to incorporating behaviour change theories and techniques in tools/toolkits in the reviewed studies. Card-sets have been found to be a helpful tool for designers; however, we argue the format of the tools for behaviour change design should not be limited to the card-set. It has been demonstrated that the qualities (e.g., likeliness, complexity, implications) of the design concepts are influenced by the materiality of the toolkit [51]. Hence, the checklist is not limited to the design of a card-set, and we intentionally phrase the questions and recommendations in the checklist so that they cover broader features. Yet, we do acknowledge that many suggestions were taken from the development of existing tools, which were mainly in the form of a card-set. Future researchers are encouraged to use generic co-design materials (e.g., LEGO, Play-doh) to support 2D tools such as card-sets and canvases during a co-design session.

### 4.4. Design Implications for Toolkit Development

Despite the judges unilaterally giving the highest score to team 3, which is the team that used the toolkit most thoroughly, we cannot conclude that the usage of the toolkit leads to a more desirable design. One possible confounding factor could be related to the knowledge and motivations of the team members. The members of team 3 appeared to be more experienced and motivated with regard to design for behaviour change and co-design with OA; therefore, they had less difficulty using the toolkit and developing the most desirable concept. A larger sample size than the current study is needed to explore this further. According to Roy and Warren [52], more empirical evidence on the assumption that design tools can support knowledge transfer during the design process is urgently needed. We contribute to this evidence base and provide insights on developing design toolkits for co-designing community-based active ageing.

Regarding toolkit development, more work needs to be done on simplifying the toolkit. Similar to what was reported by Konstanti et al. [31], participants frequently found some of the definitions and terms in BCT confusing. A side effect of the toolkit that we would like to avoid is overwhelming the participants with a large amount of information in a short time. While most of the participants found there was too much information to process, only one participant felt that the information provided was not enough. This difference was due to the different academic backgrounds of the participants and their experiences and knowledge of designing for behaviour change and co-designing with OA. This implies that it is challenging to design toolkits that are suitable for interdisciplinary use since the needs and wishes of users from different disciplines could be very different or even conflicting. However, in the above case, we were able to find a middle ground in which the information in the toolkit has a layered architecture and users can choose the layer of detailedness based on how much information they require.

The feedback on the canvas noted that we did not clearly communicate how to use the canvas to the participants. We assumed its form and text layout (facing towards each side of the canvas) would encourage the participants to stand up, and take turns for each design stage; instead, participants found the canvas was too big and did not understand why the text was arranged “so strangely”. We should explain the design decisions to the participants first. Additionally, although we intended the participants to use the card-set for ideation (as shown on the canvas), most of the teams used it for idea development and evaluation; this led us to reflect on the “script” we created for the users when designing the toolkit. The “script” could reduce the creativity of users and we will continue to allow the participants to use this toolkit flexibly. These insights helped us to extend our toolkit development checklist: (1) use a layered design to cater for different preferences regarding the amount of information, (2) explain the format of the toolkit to the users before they start using it to avoid misunderstanding, and (3) allow users to use the toolkit in any way they wish to spark creativity.

As for toolkit evaluation, first, we are aware that the context in which the toolkit is evaluated will affect the experience of using the toolkit. In our study, the toolkit was evaluated in a fast-paced environment, and the participants reflected that they did not have enough time to use the toolkit. In addition, the students had different education levels. If the toolkit was introduced in a semester interdisciplinary course for students at the same education level, where the students have more time to read and reflect on the toolkit, the feedback we collected could be different. Therefore, we encourage future researchers to report in detail on the context in which a toolkit is evaluated when reporting toolkit evaluation. Second, we suggest giving participants the option to abandon the toolkit during the design process. Observing how the toolkit was used, if at all, has helped us to triangulate the verbal feedback of the participants. Understanding the reasons behind abandonment provides insight, and this will also ensure that the research findings have high ecological validity.

It was not surprising to see design students guiding students from other disciplines to use the toolkit because the use of such toolkits is common in the design field. Since the target users for our toolkit include people without design training, it would be interesting to see which discipline, if any, would lead the team to use the toolkit when there are no design students. Surprisingly, OA’s interaction with the toolkit was minimal. This could be because OA did not expect to learn about BCT when they came to the hackathon and/or they already had ideas and wishes to discuss. This observation coincides with that of Gooch et al. [27]. Another explanation could be that OA are a very heterogeneous group, even though we revised the toolkit based on the feedback from the OA representative before the hackathon, the OA who participated in the hackathon might have had different opinions about the toolkit in comparison to what the OA representative imagined. Co-designing the toolkit with OA early on might be a more inclusive approach, which could increase the uptake of the toolkit during co-design sessions. All the OA enjoyed the hackathon, and all of the students were satisfied with their design outcomes; thus, we posit that using the toolkit is only one “means” to achieve the “end” of co-designing for community-based active ageing.

The quantitative data revealed that participants who experienced higher enjoyment during the hackathon and the toolkit usage had a higher confidence level in designing for health behaviour change and co-designing with OA at the end of the hackathon. This could be because the toolkit offers new knowledge and/or participants gained the experience to co-design with OA on health behaviour change during the hackathon. Further investigation is needed to pinpoint the reasons. Additionally, it is not possible to claim the effectiveness of the resulting interventions in promoting active ageing. More longer-term evaluation studies need to be conducted to compare the interventions developed with and without the toolkit, and a large sample size is required to reach such conclusions.

### 4.5. Limitations

This study has two major limitations. First, only Scopus was used as the database for the rapid review. We acknowledge that more insights could be gained by extracting relevant articles from multiple databases. Yet, we argue that the rapid reviews offer a sufficient starting point given the urgent need for toolkit development. Moreover, the toolkit was only evaluated with a limited sample size during a hackathon, so it is difficult to generalise the results to other contexts. However, the design implications generated from the evaluation study could apply to the development of future toolkits to support co-designing community-based active ageing. The strength of this study is that the toolkit was built based on rapid reviews from multiple disciplines, and evaluated in an interdisciplinary setting, which addresses the urgent need for researchers and practitioners to collaborate on fostering community-based active ageing with and for OA from different perspectives.

## 5. Conclusions

In this paper, referring back to the research aims, we report on the development of a toolkit to support co-design with OA for community-based active ageing. Rapid reviews were first conducted to address the three identified gaps in the current literature; this was used as the base to create the first version of the toolkit. After a few iterations based on feedback from potential users, the toolkit was evaluated in an interdisciplinary hackathon for co-designing interventions to support group gardening. The quantitative data collected from pre–post surveys indicate that the confidence levels of students with regard to both co-designing with OA and designing health behaviour change increased. The qualitative data collected from observations and interviews revealed the areas of improvement for the toolkit and the positive experiences of OA. For future work, we will evaluate an intervention developed based on the hackathon ideas and we encourage future users of the toolkit to report on their modifications, their experiences of using it, and the effectiveness of the interventions they developed.

## Figures and Tables

**Table 1:** Summary of the three rapid review searches (n: studies included).

Goal of Review	Review Type	Search Terms	n
Identify the BCTs that are effective for OA	Rapid review of systematic reviews (because of abundant research on this topic)	(TITLE-ABS-KEY (“behavio* change technique*”) AND TITLE (“systematic review”) AND TITLE-ABS-KEY (“older adult” OR “elderly” OR “senior”)).	6
Understand how to co-design with OA communities	Rapid review (because of limited research on this topic)	(TITLE-ABS-KEY (“community-based” OR “community based”) AND TITLE-ABS-KEY (“co-design” OR “participatory design”) AND TITLE-ABS-KEY (“older adult” OR “elderly” OR “senior”)).	5
Understand how to develop design tools for behaviour change	Rapid review (because of limited research on this topic)	(TITLE (toolkit OR tool) AND TITLE (“behavio* change” OR persuasive) AND TITLE (“design”))	4

**Table 2:** Effective BCTs for OA (categorised by design goals).

Design Goal	Effective BCTs for OA
Raising awareness	Information about health consequences
Enabling	Action planning
	Problem-solving
	Goal setting
	Social support
	Restructuring the physical environment
	Demonstration of the behaviour
	Instruction on how to perform a behaviour
	Graded tasks
	Adding objects to the environment
Motivating	Feedback on behaviour
	(Follow-up) prompts/cues
	Self-monitoring of outcome(s) of behaviour
	Restructuring the physical environment
	Reward approximation
	Rewarding completion
	Situation-specific reward
	Adding objects to the environment
	Social reward
	Social support
	Material reward
	Self-reward
	Non-specific reward
Fading-out	None identified

**Table 3:** Guidelines for co-designing with OA for community-based interventions.

**Involving OA**
- Select usual meeting centres of participants in the community as the workshop site to make participants feel more comfortable - Allow informal breaks during the workshop to make the environment relaxing - Keep the length of the workshop below 3 h to avoid fatigue - Keep questions direct and simple to help participants understand the questions - Provide visual materials that all participants can relate to so as to help them connect with things that they are not familiar with - Introduce one or more examples first for technology design to help them ideate based on what current technology can do - Keep the group small (2–4 people) to allow each participant enough time to speak - Divide participants into groups based on their digital skills to help participants communicate at the same level - Divide participants into groups based on their attitude to behaviour change to help participants communicate at the same level
**Fostering intergenerational bonding**
- Emphasise the common goals that all participants share - Emphasise the different skill sets that each generation have - Emphasise the values each generation could bring to each other - Let the team divide the tasks themselves in the workshop to let each participant does the task that he/she is good at within the team - Encourage life storytelling to form trust and understanding between participants
**Connecting with local organisations**
- Determine the current roles of organisations - Assessing their potential gains - Determine the roles and tasks each organisation would like to have - Reaching consensus over the division of roles and tasks - Confirm with participants about their inputs - Understand the local context, resources, and working procedure - Create a business model to ensure all the different activities are financed and the organisations are rewarded for their efforts

**Table 4:** Checklist for developing tools for co-designing with OA on behaviour change.

**Would you like your tool to be self-explanatory?** If yes - Include an intro card, booklet, article, or website for the tool and its background If not - Organise a workshop to introduce the tool and its background to the participants
**Would you like the tool to focus on one type of behaviour (e.g., physical activity)?** If yes - Provide examples that are focused on the target behaviour in the tool - Give a briefing in the tool to let participants focus on the target behaviour If not - Provide examples that cover different behaviours in the tool - Create an exercise in the tool for participants to identify the behaviour that they want to target
**Have you ensured the accessibility of your tool?** - Create a digital version that allows any people to download and print - Print on PVC, which is easy to clean and durable to use
**Have you ensured that the tool is engaging and easy to use?** - Use categories to divide the information into digestible chunks - Use categories to guide the thinking process of participants - Give a clear and concise definition and explanation for each category and its relationship with behaviour change - Give examples for each behaviour change theory/technique - Visually illustrate these examples - Use simple sentences and no jargon to explain the theory/technique - Transform guidelines into colloquial questions
**Have you ensured that OA could use the tool?** - Can they see the text? - Can they distinguish the colours used in colour coding? - Can they associate with all the illustrations given in the tool? - Can they manipulate the tool easily (e.g., separate two cards from each other)? - Can the tool communicate with them in a different way other than visually? - Can they associate with all the examples given in the tool?
**Have you associated your tool with other existing tools and toolkits?** - Provide a reference card or booklet
**Have you let participants think about what the threats to the behaviour change could be?** - Provide questions and examples to provoke this topic - Provide the opportunity for participants to tailor the tool (e.g., leave space on the cards for participants to add notes on the threats they think of)
**Have you gained feedback about your tool from the target participants during its development?** - Place the information that participants find useful in the most obvious place (e.g., in front of the cards)
**Have you conducted a pilot test of your tool with target participants?** - Do they only look at some parts of the tool (e.g., one side of the cards)? - Do they ask lots of questions or show confusion when using the tool? - Do they abandon the tool later in the design process? - Find the reasons behind these questions

**Table 5:** Questions involved in the hypothesis testing.

Question Abbreviation	Full Question in Survey
Enjoyment_All	To what extent did you enjoy the hackathon?
Expressive	I was able to be very expressive and creative while using the tools.
Concentration	While I was doing the activity (e.g., idea generation, evaluation), I was able to concentrate on the activity rather than on how to use the tools.
Enjoyed_Tools	I enjoyed the tools and would use them again.
Ease_Collaboration	I was able to work together with others easily while doing the activities.
Ease_Ideas	It was easy for me to explore many different ideas.

**Table 6:** Correlates of confidence level in co-design with OA.

Question Theme	t Value	Pr (>|t|)
Enjoyment_All	2.684	0.0436
Expressive	0.490	0.6447
Concentration	−1.209	0.2808
Enjoyed_Tools	−3.062	0.0280
Ease_Collaboration	−1.643	0.1613
Ease_Ideas	1.561	0.1793

## Data Availability

The data presented in this study are available on request from the corresponding author. The data are not publicly available due to ethical reasons.

## References

[1] Costa A., Câmara G., de Arriaga M.T., Nogueira P., Miguel J.P. (2021). Active and Healthy Aging After COVID-19 Pandemic in Portugal and Other European Countries: Time to Rethink Strategies and Foster Action. Front. Public Health.

[2] World Health Organization (2002). Active Ageing: A Policy Framework. http://www.who.int/hpr/.

[3] Boudiny K. (2013). ‘Active ageing’: From empty rhetoric to effective policy tool. Ageing Soc..

[4] Axon S. (2016). ‘The Good Life’: Engaging the public with community-based carbon reduction strategies. Environ. Sci. Policy.

[5] Verplanken B., Roy D. (2016). Empowering interventions to promote sustainable lifestyles: Testing the habit discontinuity hypothesis in a field experiment. J. Environ. Psychol..

[6] Axon S., Morrissey J., Aiesha R., Hillman J., Revez A., Lennon B., Salel M., Dunphy N., Boo E. (2018). The human factor: Classification of European community-based behaviour change initiatives. J. Clean. Prod..

[7] Michie S., Atkins L., West R. (2014). The Behaviour Change Wheel: A Guide to Designing Interventions.

[8] Ferguson H.J., Brunsdon V.E.A., Bradford E.E.F. (2021). The developmental trajectories of executive function from adolescence to old age. Sci. Rep..

[9] Castro P.C., Romano L.B., Frohlich D., Lorenzi L.J., Campos L.B., Paixão A., Deutekom M., Krose B., Dourado Z.V., de Oliveira Gomes G.A. (2020). Tailoring digital apps to support active ageing in a low income community. PLoS ONE.

[10] Brandt E., Binder T., Malmborg L., Sokoler T. Communities of everyday practice and situated elderliness as an approach to co-design for senior interaction. Proceedings of the 22nd Conference of the Computer-Human Interaction Special Interest Group of Australia on Computer-Human Interaction—OZCHI’10.

[11] APeine, Faulkner A., Jæger B., Moors E. (2015). Science, technology and the ‘grand challenge’ of ageing—Understanding the socio-material constitution of later life. Technol. Forecast. Soc. Change.

[12] Sanders E.B.-N., Stappers P.J. (2008). Co-creation and the new landscapes of design. CoDesign.

[13] Niedderer K., Clune S., Ludden G.D.S. (2017). Design for Behaviour Change.

[14] Dias S., Gama A., Maia A.C., Marques M.J., Campos Fernandes A., Goes A.R., Loureiro I., Osborne R.H. (2021). Migrant Communities at the Center in Co-design of Health Literacy-Based Innovative Solutions for Non-communicable Diseases Prevention and Risk Reduction: Application of the OPtimising HEalth LIteracy and Access (Ophelia) Process. Front. Public Health.

[15] Cheng C., Elsworth G.R., Osborne R.H. (2020). Co-designing eHealth and Equity Solutions: Application of the Ophelia (Optimizing Health Literacy and Access) Process. Front. Public Health.

[16] Bardaro G., Antonini A., Motta E. (2022). Robots for Elderly Care in the Home: A Landscape Analysis and Co-Design Toolkit. Int. J. Soc. Robot..

[17] Pedell S., Borda A., Keirnan A., Aimers N. (2021). Combining the Digital, Social and Physical Layer to Create Age-Friendly Cities and Communities. Int. J. Environ. Res. Public Health.

[18] O’Brien J., Mason A., Cassarino M., Chan J., Setti A. (2021). Older Women’s Experiences of a Community-Led Walking Programme Using Activity Trackers. Int. J. Environ. Res. Public Health.

[19] Blackwell R.W.n., Lowton K., Robert G., Grudzen C., Grocott P. (2017). Using Experience-based Co-design with older patients, their families and staff to improve palliative care experiences in the Emergency Department: A reflective critique on the process and outcomes. Int. J. Nurs. Stud..

[20] Pedell S., Keirnan A., Friday G., Miller T., Mendoza A., Lopez-Lorca A., Sterling L. (2017). Methods for Supporting Older Users in Communicating Their Emotions at Different Phases of a Living Lab Project. Technol. Innov. Manag. Rev..

[21] Keirnan A., Strachan M., Engeler B. Technology around the Park: Applying Co-Design to Resolve Conflict in Retirement Villages. Proceedings of the 31st Australian Conference on Human-Computer-Interaction.

[22] Petsani D., Mantziari D., Zilidou V., Konstantinidis E.I., Billis A., Timoleon M., Kiriakidis N., Nikolaidou M., Bamidis P.D. Co-design the future CAPTAIN system with older adults: Focusing on the e-coaching dimensions. Proceedings of the 12th PErvasive Technologies Related to Assistive Environments Conference.

[23] EBCD: Experience-Based Co-Design Toolkit—Point of Care Foundation. https://www.pointofcarefoundation.org.uk/resource/experience-based-co-design-ebcd-toolkit/.

[24] de Roeck D., Stappers P.J., Standaert A. Gearing up! A designer-focused evaluation of ideation tools for connected products. Proceedings of the NordiCHI 2014: The 8th Nordic Conference on Human-Computer Interaction: Fun, Fast, Foundational.

[25] Ambe A.H., Brereton M., Soro A., Chai M.Z., Buys L., Roe P. Older people inventing their personal internet of things with the IoT un-kit experience. Proceedings of the Conference on Human Factors in Computing Systems—Proceedings.

[26] Berger A., Odom W., Storz M., Bischof A., Kurze A., Hornecker E. The inflatable cat: Idiosyncratic ideation of smart objects for the home. Proceedings of the Conference on Human Factors in Computing Systems—Proceedings.

[27] Gooch D., Price B.A., Klis-Davies A., Webb J. (2021). A Design Exploration of Health-Related Community Displays. Proceedings of the ACM on Human-Computer Interaction.

[28] Cerna K., Müller C. Fostering digital literacy through a mobile demo-kit development: Co-designing didactic prototypes with older adults. Proceedings of the Extended Abstracts of MobileHCI 2021—ACM International Conference on Mobile Human-Computer Interaction: Mobile Apart.

[29] Güldenpfennig F. Tailor-made accessible computers: An interactive toolkit for iterative Co-design. Proceedings of the 12th International Conference on Tangible, Embedded, and Embodied Interaction.

[30] Hermsen S., Renes R., Frost J. (2014). Persuasive by Design: A Model and Toolkit for Designing Evidence-based Interventions. Creat. Differ..

[31] Konstanti C., Karapanos E., Markopoulos P. (2022). The Behavior Change Design Cards: A Design Support Tool for Theoretically-Grounded Design of Behavior Change Technologies. Int. J. Hum. Comput. Interact..

[32] Ren X., Lu Y., Oinas-Kukkonen H., Brombacher A. (2017). Perswedo: Introducing persuasive principles into the creative design process through a design card-set. Lecture Notes in Computer Science (including subseries Lecture Notes in Artificial Intelligence and Lecture Notes in Bioinformatics).

[33] Lockton D. (2017). Design, behaviour change and the Design with Intent toolkit. Design for Behaviour Change.

[34] Wang G., Albayrak A., Molenbroek J., van der Cammen T.J.M. (2018). Non-pharmacological Interventions for People with Dementia: Design Recommendations from an Ergonomics Perspective. Proceedings of the 20th congress of the International Ergonomics Association.

[35] Harker J., Kleijnen J. (2012). What is a rapid review? A methodological exploration of rapid reviews in Health Technology Assessments. Int. J. Evid.-Based Healthc..

[36] Niedderer K., Clune S., Ludden G. (2017). Introducing models, methods and tools for design for behaviour change. Design for Behaviour Change.

[37] Wang G., Kasraian D., Valk C., Kersten A., Hurst W., Yuan L., Maranus S., Jambroes M., van Wesemael P. (2022). An interdisciplinary hackathon with older adults on community-based active ageing: Outcomes and lessons learned. International Journal of Human Computer Studies.

[38] Carroll E.A., Latulipe C. The Creativity Support Index. Proceedings of the Conference on Human Factors in Computing Systems—Proceedings.

[39] Doyle L., Brady A.M., Byrne G. (2009). An overview of mixed methods research. J. Res. Nurs..

[40] Zubala A., MacGillivray S., Frost H., Kroll T., Skelton D.A., Gavine A., Gray N.M., Toma M., Morris J. (2017). Promotion of physical activity interventions for community dwelling older adults: A systematic review of reviews. PLoS ONE.

[41] Ahmed S., Heaven A., Lawton R., Rawlings G., Sloan C., Clegg A. (2021). Behaviour change techniques in personalised care planning for older people: A systematic review. Br. J. Gen. Pract..

[42] Ester M., Eisele M., Wurz A., McDonough M.H., McNeely M., Culos-Reed S.N. (2021). Current Evidence and Directions for Future Research in eHealth Physical Activity Interventions for Adults Affected by Cancer: Systematic Review. JMIR Cancer.

[43] French D.P., Olander E.K., Chisholm A., Mc Sharry J. (2014). Which behaviour change techniques are most effective at increasing older adults’ self-efficacy and physical activity behaviour? A systematic review. Ann. Behav. Med..

[44] Gardner B., Jovicic A., Belk C., Kharicha K., Iliffe S., Manthorpe J., Goodman C., Drennan V.M., Walters K. (2017). Specifying the content of home-based health behaviour change interventions for older people with frailty or at risk of frailty: An exploratory systematic review. BMJ Open.

[45] Lara J., Evans E.H., O’Brien N., Moynihan P.J., Meyer T.D., Adamson A.J., Errington L., Sniehotta F.F., White M., Mathers J.C. (2014). Association of behaviour change techniques with effectiveness of dietary interventions among adults of retirement age: A systematic review and meta-analysis of randomised controlled trials. BMC Med..

[46] Lee J.L.C., Ho R.T.H. (2022). Engaging community-dwelling older adults as co-developers in a public outdoor exercise facilities-based physical activity education intervention: A mixed-method participatory study in Hong Kong. Health Soc. Care Communityt..

[47] Gomes C.A., Ferreira S., Gouveia T., Rito P., Morais N., Sousa B. Intergenerational Participatory Design: Contributions to the development of an App. Proceedings of the 2018 International Symposium on Computers in Education (SIIE).

[48] Senteio C.R. (2019). Promoting access to health information: A method to support older African Americans with diabetes. Aslib J. Inf. Manag..

[49] LVan Velsen L., Illario M., Jansen-Kosterink S., Crola C., Di Somma C., Colao A., Vollenbroek-Hutten M. (2015). A Community-Based, Technology-Supported Health Service for Detecting and Preventing Frailty among Older Adults: A Participatory Design Development Process. J. Aging Res..

[50] Vanvoorhis C.R.W., Morgan B.L. (2007). Understanding Power and Rules of Thumb for Determining Sample Sizes. Tutor Quant Methods Psychol..

[51] Berger A., Ambe A.H., Soro A., de Roeck D., Brereton M. The stories people tell about the home through IoT toolkits. Proceedings of the DIS 2019—Proceedings of the 2019 ACM Designing Interactive Systems Conference.

[52] Roy R., Warren J.P. (2019). Card-based design tools: A review and analysis of 155 card decks for designers and designing. Des. Stud..

